# A Game Theory Algorithm for Intra-Cluster Data Aggregation in a Vehicular *Ad Hoc* Network

**DOI:** 10.3390/s16020245

**Published:** 2016-02-19

**Authors:** Yuzhong Chen, Shining Weng, Wenzhong Guo, Naixue Xiong

**Affiliations:** 1 College of Mathematics and Computer Science, Fuzhou University, Fuzhou 350116, China; yzchen@fzu.edu.cn (Y.C.); fzuwsn@163.com (S.W.); 2 Fujian Provincial Key Laboratory of Networking Computing and Intelligent Information Processing, Fuzhou University, Fuzhou 350116, China; 3 School of Information Technology, Jiangxi University of Finance and Economics, Nanchang, China; xiongnaixue@gmail.com

**Keywords:** data aggregation, game theory, vehicular *ad hoc* network, nash equilibrium

## Abstract

Vehicular *ad hoc* networks (VANETs) have an important role in urban management and planning. The effective integration of vehicle information in VANETs is critical to traffic analysis, large-scale vehicle route planning and intelligent transportation scheduling. However, given the limitations in the precision of the output information of a single sensor and the difficulty of information sharing among various sensors in a highly dynamic VANET, effectively performing data aggregation in VANETs remains a challenge. Moreover, current studies have mainly focused on data aggregation in large-scale environments but have rarely discussed the issue of intra-cluster data aggregation in VANETs. In this study, we propose a multi-player game theory algorithm for intra-cluster data aggregation in VANETs by analyzing the competitive and cooperative relationships among sensor nodes. Several sensor-centric metrics are proposed to measure the data redundancy and stability of a cluster. We then study the utility function to achieve efficient intra-cluster data aggregation by considering both data redundancy and cluster stability. In particular, we prove the existence of a unique Nash equilibrium in the game model, and conduct extensive experiments to validate the proposed algorithm. Results demonstrate that the proposed algorithm has advantages over typical data aggregation algorithms in both accuracy and efficiency.

## 1. Introduction

Vehicular *ad hoc* networks (VANETs) have recently received considerable attention. A VANET is a special type of mobile *ad hoc* network (MANET) that consists of many traveling vehicles. The vehicles in a VANET can communicate with one another using short-range wireless communication [1]. Advancements in embedded processing, wireless networking, and flexibility in sensing area selection have made creating vehicular networks possible [2]. At present, various types of vehicles are broadly equipped with sensors. These vehicle-mounted sensors can gather and share different types of information using on-board computers or global positioning system navigators [3]. Vehicles equipped with sensors can collect, process, and aggregate data [4], which can significantly improve vehicle safety or provide information on road conditions. Thus, aggregating data for a VANET is important to achieve desirable dissemination services.

Although many studies have been conducted on data aggregation in MANETs, the distinct characteristics of VANETs, such as high-speed mobility, limited transmission range, high node density, and dynamic network topology, pose significant challenges to data aggregation in VANETs. Most messages (e.g., location information and environmental data) are periodically broadcast by each vehicle in a VANET. Thus, when many vehicles participate in the VANET, the possibility of the occurrence of wireless collisions is high. For example, in a large-scale vehicular network, if every car transmits messages to the target node without reduction, then the network faces the problem of data collision and congestion. These problems lead to huge bandwidth consumption and high communication and processing costs, which reduce the efficiency of the entire vehicular network. By contrast, wireless link breakage always occurs in VANETs because of the highly mobile environment, or worse, link reestablishment increases transmission delay and network control overhead cost. Thus, the main issue for data aggregation in VANETs is how to design an efficient data aggregation approach to aggregate data with less bandwidth consumption and high accuracy in a highly dynamic network. Current studies have mainly focused on data aggregation in large-scale environments but have rarely discussed intra-cluster data aggregation in VANETs.

Game theory provides a natural way to model the data aggregation process using sensor nodes in the same cluster. In a VANET, sensors can be modeled as individual players in a data aggregation game with appropriate strategies and utility functions. This eventually leads to a global optimum data aggregation strategy in the cluster. In this study, we propose a game theory model of intra-cluster data aggregation (MGADA) based on non-cooperative game theory by analyzing the competitive and cooperative relationships among sensor nodes in VANETs. The key contributions of this study are as follows: first, several sensor-centric metrics are developed to measure data redundancy in a cluster and cluster stability in a VANET. Second, intra-cluster data aggregation in the VANET is modeled from a game theory perspective considering both data redundancy and cluster stability. Finally, a multi-player game theoretic algorithm is designed to optimize intra-cluster data aggregation in a VANET.

The rest of this paper is organized as follows: Section 2 provides a review of the related literature. Section 3 presents the problem formulation. Section 4 describes the proposed data aggregation algorithm in detail. Section 5 discusses the experiments that were conducted to validate the effectiveness of the proposed algorithm. Section 6 draws the conclusions and discusses potential additional research directions that can be pursued in the future.

## 2. Related Works

In this section, we discuss existing techniques for data aggregation in MANETs and VANETs. Data aggregation is an important issue in MANETs and has therefore been studied for decades. Various methods, including parameter estimation, rough sets, trust establishment, game theory, and particle swarm optimization, have been applied to perform data aggregation in MANETs [3,4,5,6,7,8,9,10,11,12,13,14,15]. Energy efficiency is the most significant problem in a MANET because of its limited energy capacity. Most studies aim to minimize energy cost in data transmission to prolong the lifetime of MANETs. In contrast to MANETs, VANETs have a completely different scenario because vehicles are rich in energy resources. Data aggregation in VANETa is primarily subject to the problems of link stability, data redundancy, and data accuracy. Thus, data aggregation algorithms designed for MANETs are unsuitable for VANETs, and new solutions must be developed.

Recently, several aggregation techniques for VANETs have been proposed in the literature [16,17,18,19,20,21,22,23,24,25,26,27]. Mitra *et al*. [16] proposed a novel data aggregation algorithm based on mobile agents in dynamic traffic management systems. However, the different numbers of proxy nodes and access orders to nodes have significant effects on the performance of this algorithm. Zhao and Yang [17] used mobile nodes to gather polling-based data. The authors divided the nodes into two categories: affiliated and polling nodes. Affiliated nodes send data to polling nodes through a certain number of relay hops. Polling nodes then aggregate the data and upload them to the mobile sink. However, the problem of finding an optimal subset of polling nodes is non-deterministic polynomial hard (NP-hard). Processing time increases dramatically as the network scale increases. Wang *et al*. [18] presented an aggregation algorithm based on the node distance required to send data from cluster members to the cluster head by multi-hop transmission. However, this algorithm cannot adapt to large-scale VANET scenarios because of excessive communication overhead. Scheuermann *et al*. [19] used infrastructure and stationary supporting units (SSUs) to assess dissemination performance based on the number of equipped vehicles on the road in city scenarios. If a small number of SSUs are installed in a city and connected with one another via some backbone network, then the entire vehicular network achieves interoperability and enhanced dissemination performance. However, the network undergoes significant difficulty in convergence. Realistic VANET applications have not been considered in the evaluation. Lochert *et al*. [20] proposed a hierarchical data aggregation algorithm. However, delay time evidently increases as the network scale increases. Wischhof *et al*. [21] proposed a method called segment-oriented data abstraction and dissemination to disseminate information. However, this method is unsuitable for applications that require precise aggregation results. Dietzel *et al*. [22] presented a bandwidth-efficient integrity protection mechanism for traffic efficiency application in VANETs, which uses HyperLogLog estimators to create bandwidth-efficient integrity proofs. [27] presented a generic architecture and used it to categorize different aggregations and assess their suitability to solve particular challenges. In general, most of the aforementioned data aggregation methods focus on data aggregation in large-scale environments, whereas the intra-cluster data aggregation issue is hardly addressed or explored.

Recently, the game theoretic mechanism has been extensively investigated for distributed decision-making in wireless networks. A variety of applications exist for game processing, such as power control [28,29,30], routing [8,31,32], data gathering [13,14], bandwidth sharing [33], congestion control [34], and topology control [35] in MANETs. In [13], an evolutionary game-based data aggregation model (EGDAM) in wireless sensor networks was proposed, and an evolutionary game-based adaptive weighting algorithm (EGWDA) was provided for pixel-level data aggregation with homogeneous sensors. In [14], the interaction between sensors and monitoring nodes was formulated as a dynamic game with incomplete information, and a reputation system was built to ensure reliable data fusion by confining the fusion process to trustworthy sensors. Thus, the game theory mechanism is suitable to build effective functions for decision-making processes. However, few studies have investigated data aggregation in dynamic VANETs. Existing algorithms for MANETs cannot be directly applied to VANETs because of their different features in terms of node characteristics (e.g., high power, quick motion, and low bandwidth).

## 3. Problem Statement

In VANETs, we divide the data aggregation process into three stages: cluster initialization stage, cluster stabilization stage, and cluster reconstruction stage. In the first stage, the cluster initialization stage, all the nodes in the vehicular network are grouped into several temporary clusters. A cluster head is then selected for each temporary cluster. The main goal of this stage is to find a reasonable mechanism to select a cluster head and facilitate intra-cluster communication.

In the second stage, the cluster stabilization stage, the temporary cluster is relatively stable. The nodes in the temporary cluster send sample data to the cluster head. During this stage, each sending node generates a data report in each sampling period. The information in a data report contains the node location, sample data, sample quality, and the sequence of the sampling period.

In the second stage, the first challenge is to estimate the sample quality of each node. The second challenge is to construct a stable cluster for data aggregation in highly mobile vehicular networks that can reduce packet loss and improve communication quality. The third challenge is to find an optimal transmission strategy for efficient data aggregation, which can be formulated as shown below.

Supposing that there are *n* nodes in a cluster, and each node independently samples data, then *p_i_*^(*k*)^ is the transmission strategy of node *i* in the *k*-th sampling period, which is defined as follows:

(1) $$p_{i}^{\left(k\right)}=\{\begin{array}{c} 0\\ 1 \end{array}$$

If $p_{i}^{\left(k\right)}=1$, then node *i* is a sending node that sends data report to the cluster head in the *k*-th sampling period. If $p_{i}^{\left(k\right)}=0$, then node *i* is a candidate node that remains silent in the *k*-th sampling period. Thus, the problem in this step is to find an optimal combination of transmission strategy, $\left\{p_{1}^{\left(k\right)},p_{2}^{\left(k\right)},p_{3}^{\left(k\right)},...,p_{n}^{\left(k\right)}\right\}$, which can reduce the number of sending nodes to improve transmission efficiency while preserving data aggregation accuracy.

The third stage is the cluster reconstruction stage. During the cluster stabilization stage, the cluster stability is monitored continuously. If the metric for evaluating the cluster stability exceeds a predefined threshold, the cluster is considered broken. Consequently, the process enters the cluster reconstruction stage, and the cluster is reconstructed by reentering the cluster initialization stage.

To solve the aforementioned challenges, we design several metrics to analyze data redundancy in VANETs by estimating the sample quality of each node. Given that the rapid motion of vehicles results in frequent and dramatic topological changes in VANETs, we also propose several metrics to estimate cluster stability in VANETs. By considering cluster stability, MGADA can improve communication quality and data transmission quality in the data aggregation process. Finally, to find an optimal combination of transmission strategy, we study the competition and cooperation relationships among the sensor nodes involved in data aggregation based on game theory, and employ Nash equilibrium and interruption processing to optimize the transmission strategy in VANETs.

## 4. MGADA

### 4.1. Cluster Initialization

Cluster stability is very important for data aggregation in the highly dynamic VANET. We introduce a typical clustering algorithm named position-based prioritized clustering [36] for cluster head selection in the cluster initialization stage. In this clustering algorithm, the relative movement in the neighborhood, leadership duration, and moving direction are considered for clustering. Each node broadcasts beacons to announce itself to be a cluster head. The beacon contains the number of clock periods, node ID, moving direction, node location, and leadership. When a node receives a beacon from a neighbor, it compares the neighbor’s moving direction with its own. If they have the same direction, then the node joins the cluster its neighbor belongs to when the following three conditions are satisfied: its neighbor is the cluster head; its neighbor has a larger leadership than it has; and its neighbor has a smaller ID number than it has.

### 4.2. Cluster Stabilization Stage

#### 4.2.1. Sample Quality Estimation

In this subsection, we introduce several definitions to address the problem of estimating the sample quality of a node:

Sample Quality. In general, the sample quality of a node depends on both real-time and historical sample data. We can evaluate the sample quality of one node by calculating the deviation among the sample data in a sliding window, which can reflect the extent of the variation in the sample data in a particular time interval. Thus, the sample quality can be defined as follows:

(2) $$SQ_{x}^{\left(k\right)}=\frac{1-\alpha \sigma_{x}^{2}\left(k\right)}{{\left(DR_{x}^{\left(k\right)}-\bar{DR_{x}^{\left(k\right)}}\right)}^{2}}$$

(3) $$\sigma_{x}^{2}\left(k\right)=std\left(DR_{x}^{\left(q\right)},DR_{x}^{\left(q+1\right)},...,DR_{x}^{\left(k\right)}\right)$$

(4) $$\bar{DR_{x}^{\left(k\right)}}=\frac{1}{k-q+1}\sum_{i=q}^{k}DR_{x}^{\left(i\right)}$$

(5) $$q=\{\begin{array}{c} 1,k\leq w\\ k-w+1,k>w \end{array}$$

where $SQ_{x}^{\left(k\right)}$ denotes the sample quality of node *x* in the *k*-th sampling period, $DR_{x}^{\left(k\right)}$ is the data sampled by node *x* in the *k*-th sampling period, *q* is the sequence number of the sampling period at the starting position of the sliding window, *w* is the size of the sliding window, and *a* is the adjustment factor and ranges from 0 to 1. $\bar{DR_{x}^{\left(k\right)}}$ and $\sigma_{x}^{2}\left(k\right)$ denote the mean value and variance, respectively, of the sample data set collected by node *x* during the sampling periods that fall within the sliding window.

Calculating $SQ_{x}^{\left(k\right)}$ requires the mean and variance values of the entire sample data collected in the sliding window. Given the constraints of storage resources and the processing capacity of the nodes, buffering all the sample data in the sliding window is impractical. However, we can simplify the calculation of $SQ_{x}^{\left(k\right)}$ by storing the intermediate results of $DR_{x}^{\left(k-1\right)}$:

(6) $$\bar{DR_{x}^{\left(k\right)}}=\left\{ \begin{array}{l} \frac{1}{k}\left(\left(k-1\right)\times \bar{DR_{x}^{\left(k-1\right)}}+DR_{x}^{\left(k\right)}\right),k\leq w\\ \frac{1}{w}\left(w\times \bar{DR_{x}^{\left(k-1\right)}}+DR_{x}^{\left(k\right)}-DR_{x}^{\left(k-w\right)}\right),k>w \end{array}\right.$$

Here we provide a brief explanation of Equation (6). When the sequence number of the current sampling period, *k,* is less than the size of the sliding window, *w*, that is, k≤w, then all the data collected by node *x* falls into the sliding window, so that $\bar{DR_{x}^{\left(k\right)}}$ can be obtained by adding $DR_{x}^{\left(k\right)}$ to $\left(k-1\right)\times \bar{DR_{x}^{\left(k-1\right)}}$, which is the sum of the data collected in the previous *k* − 1 sampling periods. In another case, when the sequence number of the current sampling period, *k,* is larger than the size of the sliding window, *w*, that is, k>w, $\bar{DR_{x}^{\left(k\right)}}$ is the mean value of the data set {$DR_{x}^{\left(k-w+1\right)},DR_{x}^{\left(k-w+2\right)},...,DR_{x}^{\left(k-1\right)},DR_{x}^{\left(k\right)}$}, and $\bar{DR_{x}^{\left(k-1\right)}}$ is the mean value of the data set {$DR_{x}^{\left(k-w\right)},DR_{x}^{\left(k-w+1\right)},...,DR_{x}^{\left(k-1\right)}$}, so that $\bar{DR_{x}^{\left(k\right)}}$ can be obtained by adding $DR_{x}^{\left(k\right)}$ to $w\times \bar{DR_{x}^{\left(k-1\right)}}$ and subtracting $DR_{x}^{\left(k-w\right)}$ from $w\times \bar{DR_{x}^{\left(k-1\right)}}$, which is the sum of {$DR_{x}^{\left(k-w\right)},DR_{x}^{\left(k-w+1\right)},...,DR_{x}^{\left(k-1\right)}$}.

To analyze the distribution of the sample quality in a cluster, $SQ_{x}^{\left(k\right)}$ must be normalized. We use the linear normalization function to map the sample quality value within the range of [0, 1] as follows:

(7) $${\left(SQ_{x}^{\left(k\right)}\right)}^{'}=\frac{SQ_{x}^{\left(k\right)}-Min\left(SQ^{\left(k\right)}\right)}{Max\left(SQ^{\left(k\right)}\right)-Min\left(SQ^{\left(k\right)}\right)}$$

where $Max\left(SQ^{\left(k\right)}\right)$ and $Min\left(SQ^{\left(k\right)}\right)$ denote the maximum and minimum sample quality, respectively, in the neighborhood set of node *x* in the *k*-th sampling period.

Mutual Quality Gain. To compare the sample quality of adjacent nodes, we define the mutual quality gain, which can evaluate sample quality distinction between nodes *y* and *x* in the *k*-th sampling period as follows:

(8) $$MQG_{xy}^{\left(k\right)}=Max\left(\log_{10}\frac{SQ_{x}^{\left(k\right)}}{SQ_{y}^{\left(k\right)}},0\right)$$

where $SQ_{x}^{\left(k\right)}$ and $SQ_{y}^{\left(k\right)}$ represent the sampling quality of nodes *x* and *y* in the *k*-th sampling period, respectively. A large difference in the sample quality of nodes *x* and *y* corresponds to a high mutual quality gain. Furthermore, with the introduction of a logarithmic function, if node *x* has a higher sample quality than its neighbor node *y*, then Equation (8) has a positive value. Otherwise, Equation (8) has a negative value. To conveniently estimate the sample quality of a node in its neighborhood, the negative gain is adjusted to 0. Hence, Equation (8) can clearly distinguish between the sample qualities of adjacent nodes and enhance the mutual quality gain of the nodes whose sample qualities are relatively high in their neighbor domain.

Neighborhood Retroaction Quality. Neighborhood retroaction quality is defined to estimate the relative sample quality of a node in its neighborhood. If the neighborhood retroaction quality of a node is low, then its sample quality is relatively low in its neighbor domain, and its sample data can be reduced to improve transmission efficiency. The neighborhood retroaction quality of node *x* in the *k*-th sampling period $NRQ_{x}^{\left(k\right)}$ is denoted as follows:

(9) $$NRQ_{x}^{\left(k\right)}=\sum_{y\in N_{x}^{\left(k\right)}}MQG_{xy}^{\left(k\right)}$$

(10) $${\left(NRQ_{x}^{\left(k\right)}\right)}^{'}=\frac{NRQ_{x}^{\left(k\right)}-Min\left(NRQ^{\left(k\right)}\right)}{Max\left(NRQ^{\left(k\right)}\right)-Min\left(NRQ^{\left(k\right)}\right)}$$

where $MQG_{xy}^{\left(k\right)}$ is the mutual quality gain from nodes *y* to *x* in the *k*-th sampling period. ${\left(NRQ_{x}^{\left(k\right)}\right)}^{'}$ is the normalized neighborhood retroaction quality of each node.

**Figure 1:**
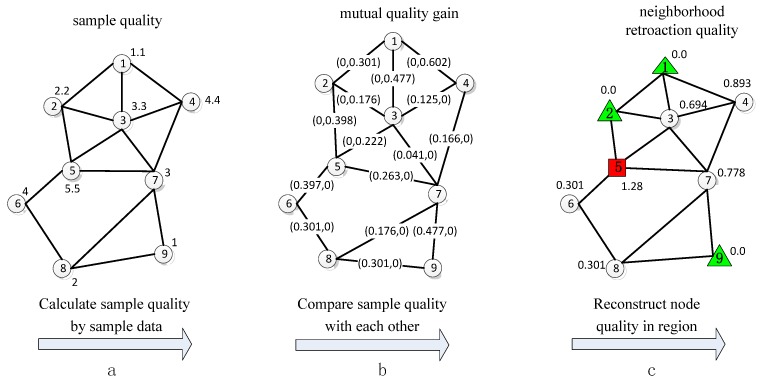
Example illustrating the three stages of estimating node sample quality.(**a**) calculate sample quality; (**b**) calculate mutual quality gain; (**c**) calculate neighboord retroaction quality.

Figure 1 illustrates the process of estimating the sample quality of each node in a cluster with nine nodes. First, as shown in Figure 1a, each node calculates its sample quality according to its sample data. Second, as shown in Figure 1b, each node acquires the sample quality of its neighbor nodes through message broadcasting and then achieves mutual quality gain by comparing its sample quality with those of its neighbor nodes. Two values exist between each node pair in Figure 1b. The first value corresponds to the mutual quality gain of the node with a higher node ID from the node with a lower node ID. The second value represents the mutual quality gain in the opposite direction. Finally, as shown in Figure 1c, each node obtains its neighborhood retroaction quality by summing up all the mutual quality gains of its neighbor nodes. For example, node 5 has the highest neighborhood retroaction quality in its cluster; hence, it is likely to be chosen as the sending node. By contrast, nodes 1, 2, and 9 have the lowest neighborhood retroaction quality in their clusters and are assumed as the candidate nodes.

Cluster Redundancy Degree. Cluster redundancy degree $CRD_{P}^{\left(k\right)}$ is utilized to evaluate the redundancy degree of the sample data collected in a cluster in the *k*-th sampling period when adopting a given transmission strategy profile *P*, which is defined as follows:

(11) $$CRD_{P}^{\left(k\right)}=\frac{1}{\left|C\right|}\left(|C|-\sum_{i\in C}p_{i}^{\left(k\right)}\times NRQ_{i}^{\left(k\right)}\right)$$

where *C* denotes the node set of a cluster, $p_{i}^{\left(k\right)}$ is the transmission strategy of cluster member *i* in the *k*-th sampling period, $P=\{p_{i}^{\left(k\right)}|i\in C\}$ denotes the transmission strategy set of cluster *C* in the *k*-th sampling period, and $NRQ_{i}^{\left(k\right)}$ is the neighborhood retroaction quality of node *i* in the *k*-th sampling period.

#### 4.2.2. Cluster Stability Estimation

Given the high-speed mobility of vehicles, the topological structure of VANETs varies frequently, which significantly affects data aggregation quality. To guarantee data aggregation quality, we introduce two parameters: separation vector gain and cluster variation degree, to analyze link stability and cluster stability, respectively:

Separation Vector Gain. The separation vector gain is designed to evaluate the link stability between adjacent nodes. If the relative displacement among adjacent nodes increases, then the link stability becomes weak, and vice versa [37]. Based on this condition, the separation vector gain is defined as follows:

(12) $$SVG_{v_{i}}^{\left(k\right)}\left(v_{j}\right)=Max\left(10\mathrm{lg}\frac{D^{\left(k\right)}\left(v_{j}\to v_{i}\right)}{D^{\left(k-1\right)}\left(v_{j}\to v_{i}\right)},0\right)$$

where *v_i_* and *v_j_* denote the receiving and sending nodes, respectively. $D^{\left(k\right)}\left(v_{j}\to v_{i}\right)$ denotes the relative displacement between *v_i_* and *v_j_* in the *k*-th sampling period, and $D^{\left(k-1\right)}\left(v_{j}\to v_{i}\right)$ denotes the relative displacement between *v_i_* and *v_j_* in the previous sampling period.

**Figure 2:**
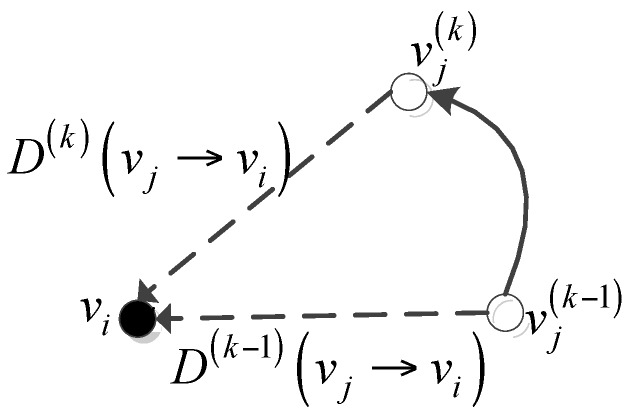
Diagram of separation vector gain.

Equation (12) evaluates the link stability by analyzing the variation tendency of the relative displacement between two adjacent nodes. A large separation vector gain indicates that the link is prone to instability, and vice versa. As shown in Figure 2, if nodes *v_i_* and *v_j_* are moving away from each other, then the signal strength of the wireless link between them decreases, which indicates that the probability of link failure increases. On the contrary, if nodes *v_i_* and *v_j_* are moving toward each other, then the signal strength of the wireless link between them, which indicates that the wireless link is likely to be stable.

Cluster Variation Degree. The cluster variation degree is designed to evaluate the variation tendency of cluster stability on a timeline and is defined as follows:

(13) $$CVD_{H}^{\left(k\right)}=\frac{1}{k-q+1}\sum_{i=q}^{k}std\left(SVG_{H}^{\left(i\right)}\left(y\right)\right),y\in C_{H}$$

where $CVD_{H}^{\left(k\right)}$ denotes the cluster variation degree of the cluster $C_{H}$, whose cluster head is node *H* in the *k*-th sampling period; $SVG_{H}^{\left(i\right)}$ is the separation vector gain between cluster member *y* and cluster head *H* in the *i*-th sampling period; and *q* denotes the sequence of the sampling period at the start position of the sliding window, which is described in Equation (5). *std* indicates the standard deviation function of the separation vector gain. A small cluster variation degree indicates that the cluster is likely to be stable. Furthermore, considering the historical information of link stability, we can reduce the effects of the abnormal behavior of individual nodes on estimating cluster stability. The detail of the algorithm for estimating sample quality and cluster stability is listed as Algorithm 1.

**Algorithm 1.** Estimation of Sample Quality and Cluster Stability1:
*Procedure Estimation*
2:*k* ← 13:
*for k=1 to Number_of_Sampling_Period do*
4:
*For for each x∈ V*
5:
*Get sample data in k-th sampling period*
6:
*Get x’s position in k-th sampling period*
7:
*Update sliding window*
8:
$SQ_{k}\leftarrow 1-\alpha \sigma_{x}^{2}\left(k\right)/{\left(DR_{x}^{\left(k\right)}-\bar{DR_{x}^{\left(k\right)}}\right)}^{2}$
9:
*Broadcast message that contains sample data, position, SQ_k_, node id*
10:
$MQG_{k}\leftarrow Max\left(\log_{10}SQ_{x}^{\left(k\right)}/SQ_{y}^{\left(k\right)},0\right),\;\forall y\in N_{x}^{\left(k\right)}$
11:
$NRQ_{x}[k]\leftarrow \sum_{y\in N_{x}^{\left(k\right)}}MQG_{xy}^{\left(k\right)}$
12:
$SVG_{xy}\left[k\right]\leftarrow Max\left(10\mathrm{lg}D^{\left(k\right)}\left(y\to x\right)/D^{\left(k-1\right)}\left(y\to x\right),0\right),\forall y\in C\left(x\right)$
13:
*Send message (NRQ_x_*[*k*], *SVG_k_*, *x*, *k ) to cluster head*
14:
*end for*
15:
*k++*
16:
*end for*
17: 
*end procedure*


#### 4.2.3. Game Formulation of Data Aggregation

In this subsection, we describe the game model, including players, strategies, and the utility function. The utility function is designed by studying the competition and cooperation relationships among vehicles. We also prove that there exists a unique Nash equilibrium that corresponds to the optimal transmission strategy.

##### (1) Multi-Player Game Model

A game of intra-cluster data aggregation in VANET is an interactive decision-making process between a set of self-interested nodes, which formally consists of the following elements:

*I*: In a VANET, a set of players, individual vehicles, or wireless sensor nodes in a cluster is considered to be the players in the game. The number of players in player set *I* is *n*.

*p_i_*: A set of strategies that is viable for the player to make a decision. In a VANET, the strategy of player *i* in the *k*-*th* sampling period is a binary value $p_{i}^{\left(k\right)}\in \left\{0,1\right\}$, where $p_{i}^{\left(k\right)}=1(p_{i}^{\left(k\right)}=0)$ represents the choice of player *i* of sending/not sending a data report to cluster head *H*. Strategy profile, *P,* is a set of transmission strategies chosen by *n* nodes, that is, {$p_{1}^{\left(k\right)},p_{2}^{\left(k\right)},p_{3}^{\left(k\right)},...,p_{n}^{\left(k\right)}$}. We represent the strategy profile with *P* = {*p_i_*, *p_−i_*}, where *p_i_* is the strategy of player *i*, and *p_−i_* denotes the transmission strategy chosen by other n−1 cluster members.

*u_i_*: The payoff, utility function, or benefit of player *i* when adopting strategy profile *P*. In a VANET, the utility function represents the performance of data aggregation in a cluster.

The game of intra-cluster data aggregation in a VANET is to determine an optimal transmission strategy profile, *P* = {$p_{1}^{\left(k\right)},p_{2}^{\left(k\right)},p_{3}^{\left(k\right)},...,p_{n}^{\left(k\right)}$} = {*p_i_*, *p_−i_*}, to achieve maximal utility. Each cluster member sends its own information of neighborhood retroaction quality and separation vector gain to the cluster head and competes with other cluster members in a sending node to optimize the utility function. In a VANET, we mainly focus on two aspects of achievable utility: cluster-level data redundancy (*i.e*., represented by the cluster redundancy degree) and cluster stability (*i.e*., represented by the cluster variation degree). Hence, the utility function of the non-cooperative data aggregation game is defined as the linear combination of the cluster redundancy degree and cluster variation degree, that is:

(14) $$\{\begin{array}{c} U\left(P,k\right)=-\tau \times CRD_{P}^{\left(T_{2}\right)}+\left(\tau -1\right)\times CVD_{H}^{\left(T_{2}\right)}\\ \arg\max\underset{p_{i}^{\left(T_{2}\right)}\in \left\{0,1\right\}}{U\left(P,k\right)}\;P=\left\{p_{1}^{\left(T_{2}\right)},p_{2}^{\left(T_{2}\right)},...,p_{n}^{\left(T_{2}\right)}\right\}\; \end{array}$$

where τ is the regulatory factor ranging from 0 to 1, which is used to adjust the effect of data redundancy and cluster stability on the utility function. *T_2_* is the time the game processing lasts. The most common solution for a non-cooperative strategic form game is Nash equilibrium. In a non-cooperative game, each cluster member is assumed as a fully rational entity. Each entity intends to send its own data report to the cluster head while reducing data redundancy and enhancing cluster stability. The entire cluster achieves Nash equilibrium when the nodes can no longer increase their utility through individual effort.

##### (2) Nash Equilibrium

In game theory, a game may possess a large number of equilibrium or none at all. Before the derivation of the multi-player game theoretic algorithm for intra-cluster data aggregation (MGADA) for the non-cooperative data aggregation game, we must prove that a unique Nash equilibrium solution exists in the proposed data aggregation scheme.

**Lemma 1.**  *A cluster transmission strategy, P = {*$p_{1},p_{2},p_{3},...,p_{n}$*}, is an NE of [N, {*$p_{i}$*}, {u_i_(^.^)}], for every* i∈I *and* $p_{i}^{'}\in P$*. If* $u_{i}\left(p_{i},p_{-i}^{*}\right)\geq u_{i}\left(p_{i}^{'},p_{-i}^{*}\right)$*, then no node can improve its payoff through individual deviation.*

**Theorem 1.**  *A* *unique* *Nash equilibrium* *exists in game, [N,{*$p_{i}$*},{u_i_(^.^)}], if, for all i = 1,2,…,n,*

1. P is a non-empty, convex, and compact subset of the same Euclidean space R^n^.

*2. The utility function satisfies the following condition:*

(15) $$\frac{\partial^{2}f\left(p_{1},...,p_{n}\right)}{\partial p_{u}\partial p_{v}}\geq 0,u\neq v$$

That is, the utility function *f*(*P*) is a type of super modular function.

Nash equilibrium is the most widely used solution in game problems. In addition to this, the proof of Theorem 1 is given in the Appendix A. However, $p_{i}$ is strictly constrained to 0 or 1; whereas the value of $p_{i}^{*}$ may be decimal (the solution of $p_{i}^{*}$ is given in Appendix B). Under these conditions, we attempt to search for locally optimal solutions by employing the interruption process shown below.

##### (3) Interruption Process

First, $p_{i}^{*}$ solution is initialized to be the unique Nash equilibrium solution, and its decimal strategies are allowed to be 0 or 1 randomly. The interruption stage then follows. The interruption function ξ is defined below. This random number is generated by each participant *i*, that is, *R_i_*. Furthermore, the procedure is realized through the statement rand (0, 1). Second, *R_i_* is compared with the constant perturbation frequency γ, which is preset in the algorithm. If *R_i_* < γ, then one element *p_i_*ʹ in the strategy set *p* is randomly selected, and a new transmission strategy combination *P*ʹ is formed. The utility value of *f*(*P*ʹ) is measured and compared with the current optimal strategy combination *P*. If *f*(*P*ʹ) is larger than *f*(*P*), then *p_i_*ʹ is substituted for the current strategy *p_i_*; otherwise, the strategy remains unchanged. After perturbations are performed on all the participants, a new transmission strategy combination *P'* can be obtained. Finally, after several perturbation and reconstruction processes, a stable transmission strategy combination in a cluster is obtained. The detail of the game process is described in Algorithm 2.

**Algorithm 2.**
*GameProcess*1:
*Procedure GameProcess*
2:
*t ← 1*
3:
P* ← Get Nash equilibrium solution by Equation (14)
4:
R* ← 0
5:
γ*←Get Nash equilibrium solution by Equation (14)*
6:
*if P has no decimal solution then*
7:
*return*
8:
*end if*
9:
*Initialize cluster strategy P’*
10:
*for each x∈ V do*
11:
*if (p_x_ != 0 && p_x_!= 1) then*
12:
*P_x_^(t)^ ← randomly choose 0 or 1*
13:
*end if*
14:
*end for*
15:
*while the network composed by P is not stable*
16:
*for each x∈ V do*
17:
*R_x_=generate a random number*
18:
*if R_x_ < γ then*
19:
*P_x_*’ *← change node strategy*20:
*if f(P’) > f(P^(t)^) then*
21:
*P^(t+1)^ = P’*
22:
*else*
23:
*P^(t+1)^ = P^(t)^*
24:
*end if*
25:
*end if*
26: 
*end for*
27:
*end while*
28:
*end procedure*


##### (4) Protocol Description

A brief description of the protocol communication in the second stage is listed as follows:

(1) Each node collects a series of sampling data and stores them in the sliding window. Each node broadcasts its own reliability in the “Reliability” message to its neighbors. The “Reliability” message contains the following information: the number of clock period, node ID, node reliability, and node position.
(2) Each node calculates its *NRQ* and *SVG* when it receives the “Reliability” message from its neighbors. It then sends its own *NRG* and *SVG* in the “Attribute” message to the cluster head. The “Attribute” message contains the number of clock period, node ID, *NRQ*, and *SVG*.
(3) The cluster head calculates the *CRD* and *CVD* after receiving all the “Attribute” messages from its cluster members and then performs the game process to obtain the transmission strategy.
(4) After the game process, the cluster head broadcasts a “Confirm” message, which contains the node ID of the cluster members selected as the sending nodes to its cluster members. Then the sending nodes send a “Data” message, which contains the number of clock period, node ID, and the sampling value, to the cluster head.
(5) The cluster head aggregates the sampling data from the sending nodes and transmits the aggregated data to the sink node.

### 4.3. Cluster Reconstruction Stage

When the topology of a VANET changes significantly and the network structure is severely damaged, the aggregation performance in the cluster decreases significantly. Equation (12) shows that, if the separation vector gain of the link reaches $10\mathrm{lg}R_{trans}/R_{nei}$, then normal communication is broken. Moreover, if the separation vector gain in the cluster exceeds $10n\mathrm{lg}R_{trans}/R_{nei}$ (*i.e*., *n* is the number of nodes in a cluster), then the cluster structure significantly changes and becomes unsuitable for data aggregation using the old transmission strategy combination. Hence, a new aggregation sub-cluster must be reconstructed.

If a cluster enters the cluster reconstruction stage, the original cluster head broadcasts the “Reconstruction” message to its cluster members to notify that the cluster head selection process has been re-initialized.

## 5. Experiments

### 5.1. Simulation Settings

In this section, we present the simulation results to validate the performance and effectiveness of MGADA. The algorithms are implemented in the ns-2 simulator using C++. For media access, we use the original version of the standard IEEE 802.11 with a maximum transmission rate of 2 Mbps. We adopt the tow-ray ground model as the radio propagation model. The detail of the parameter settings in the simulation is shown in Table 1. All the simulations are run for 1800 s, and all the simulation results are averaged over 50 runs. We perform road infrastructure simulation using a topologically integrated geographic encoding and referencing [38] dataset provided by the United States Census Bureau. In the simulations, the random way point [39] model is applied in node motion. When a node reaches its destination, it stops for 5 s, chooses a random speed and another destination, and then moves to the destination at the chosen speed. In the experiments, the performances of the MGADA under different scenarios are compared with those of several typical algorithms, including randomized waiting (RW) [15], GLOBAL scheme, and mobile agent-based strategy (MAS) [16], in terms of network stability, data reduction, and data accuracy. The GLOBAL aggregation scheme aggregates all samples in each sampling period. RW is an application layer mechanism that introduces artificial delays and increases temporal convergence at each source node for each packet to achieve efficient data aggregation without the explicit maintenance of a structure.

Here we introduce four metrics—the stability, compression, accuracy, and overhead ratios—to evaluate the network stability, data reduction, aggregation accuracy, and overhead, respectively. The network stability is defined in Equation (14), and the compression ratio is defined as the percentage of the sending nodes. The accuracy ratio is defined as the ratio of the sample data to the baseline data, and the overhead is defined as the percentage of non-application bytes divided by the total number of bytes sent by MGADA. We also analyze the protocol overhead of MGADA and discuss the impact of utility factor on MGADA.

### 5.2. Analysis of Experimental Results

**Figure 3:**
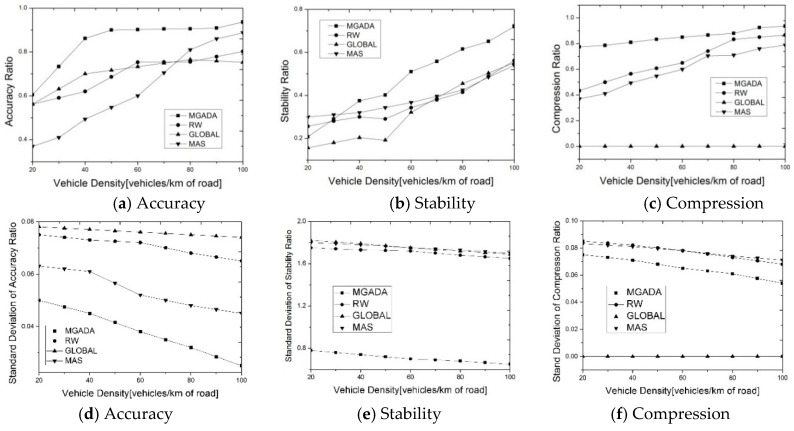
Performance with different vehicle densities.

Figure 3 indicates the impact of vehicle density on the performance in aggregation accuracy, network stability, and data reduction. In this simulation, the vehicles are moving in fixed traffic roads at a maximum velocity of 15 m/s. The directions of the vehicles are relatively restricted. Figure 3a–c show the variation of the accuracy, stability, and compression ratios with vehicle density.

In Figure 3a all the algorithms perform well in aggregation accuracy. However, MGADA can improve the aggregation accuracy much faster than the other algorithms when the vehicular density is less than 40 vehicles/km because MGADA can maintain a more stable cluster structure in a spare network than the other algorithms. In addition, when vehicle density reaches a certain degree, further improvement in aggregation accuracy with an increment in vehicle density is difficult. Figure 3a shows that aggregation accuracy remains stable over [0.9, 0.95] when vehicular density is more than 60 vehicles/km. Figure 3a also shows that the performance of RW is close to that of GLOBAL scheme. RW attempts to wait for data reports with random delays and therefore exhibits the same amount of randomness in accuracy as GLOBAL scheme. The aggregation performance in MAS is most dependent on vehicle density, compared with that in the other algorithms because of the optimized capability of mobile agents. However, MAS does not exhibit good performance in a sparse network because, in this situation, the cooperation of vehicles is weak and the mobile agent is unsuitable for aggregation. MGADA is superior to the other three algorithms under different density scenarios.

Given its effective strategy, Figure 3b shows that MGADA achieves the best performance in cluster stability among the compared algorithms, whereas the stabilities achieved by RW and MAS are the same as that of GLOBAL scheme. These findings prove that the compared algorithms do not achieve aggregation in an optimal manner. MGADA performs better than the compared algorithms because of its advantages in maintaining cluster stability by game processing.

Figure 3c demonstrates that MGADA sacrifices the compression ratio to improve aggregation accuracy. The compression ratio rises as vehicle density increases because the volume of propagation data is enormous in high density. Moreover, the required information for aggregation is limited to the known area. In a sparse network, the effective integration of data is limited. MGADA uses a self-adaptive strategy and can aggregate more data than the other algorithms. However, RW and MAS are different. In a sparse network, RW cannot obtain information adaptively using the random waiting strategy. MAS collects information using mobile agents, which adaptively choose strategies through distances and angles. In a sparse network, the connection among each vehicle is weak, and jumping from one vehicle to another is not beneficial for mobile agents. Thus, the compression ratio in MAS is close to that in RW, as shown in Figure 3c. Figure 3 shows that the aggregation scheme, which searches for a stable topology with less dependence on density, is efficient for aggregation in a VANET.

Figure 3d–f show the standard deviations of aggregation accuracy, network stability and data reduction with different vehicle densities of all the algorithms. From Figure 3d,e, we can find that MGADA not only achieve the best performance in aggregation accuracy and network stability but also the lowest standard deviation of aggregation accuracy and network stability among all the algorithms in all the simulation scenes. From Figure 3f, because GLOBAL schemes aggregate data without compression, we can find that MGADA also achieves the lowest standard deviation of compression ratio among all the algorithms except GLOBAL scheme.

**Figure 4:**
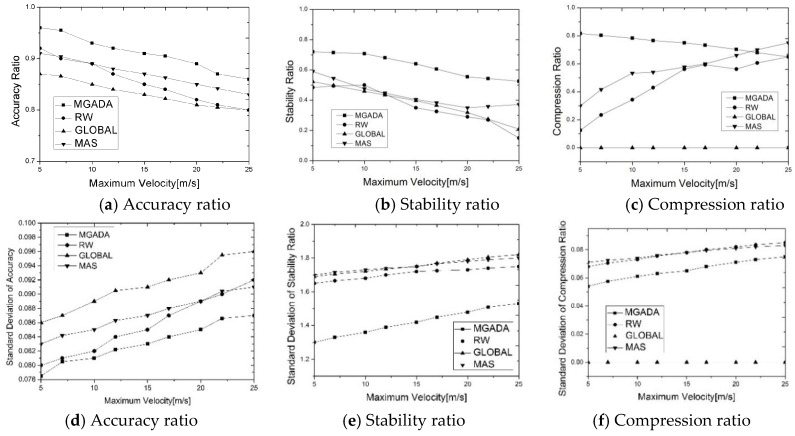
Performance with different maximum vehicle velocities.

Figure 4 shows the impact of vehicle velocity on MGADA, RW, MAS, and GLOBAL scheme. In this simulation, the vehicle density is set at 60 vehicles per kilometer of road. As shown in Figure 4a,b, all the algorithms show a decline in accuracy and stability with an increase in vehicle velocity. Rapid vehicular motion can speed up message delivery and result in considerable package losses. Moreover, as shown in Figure 4a, when the maximum velocity is over 10 m/s, the accuracy ratio decreases as maximum velocity increases. A large maximum velocity and a large number of vehicles with different velocities correspond to the high instability of the global aggregation environment. Thus, RW, MAS, and GLOBAL scheme do not perform well on the indices of stability ratio and accuracy ratio. In Figure 4a, we can also see that the accuracy ratio of MGADA stabilizes at approximately 90%, whereas those of the other three algorithms vary more and exhibit poorer performance, which proves that MGADA can achieve superior aggregation results compared with the other algorithms in a high velocity scenario.

Furthermore, a large maximum velocity can weaken link stability, which can cause the compression ratios in RW and MAS to increase with a rise in vehicle velocity, as shown in Figure 4c. However, the compression ratio in MGADA decreases with an increment in vehicular velocity, as shown in Figure 4c. Vehicles with high motion find it difficult to maintain a stable cluster structure. The higher the vehicle velocity is, the more unstable the network is. This means that more nodes are needed for well aggregation when the amount of nodes remains unchangeable. Thus, the compression ratio in MGADA decreases with an increase in vehicular velocity. The compression ratio in RW is smoother than that in MAS because of the disadvantages of mobile agents and the flexibility of random waiting for data reports. In addition, MGADA, which searches for a stable aggregation environment, is effective in a VANET.

Figure 4d–f show the standard deviations of aggregation accuracy, network stability and data reduction with different maximum vehicle velocities of all the algorithms. It’s obvious that MGADA still achieves the lowest standard deviations in all the simulation scenes. All the simulation results above demonstrate that MGADA has the advantage of fine stability over the compared algorithms.

**Figure 5:**
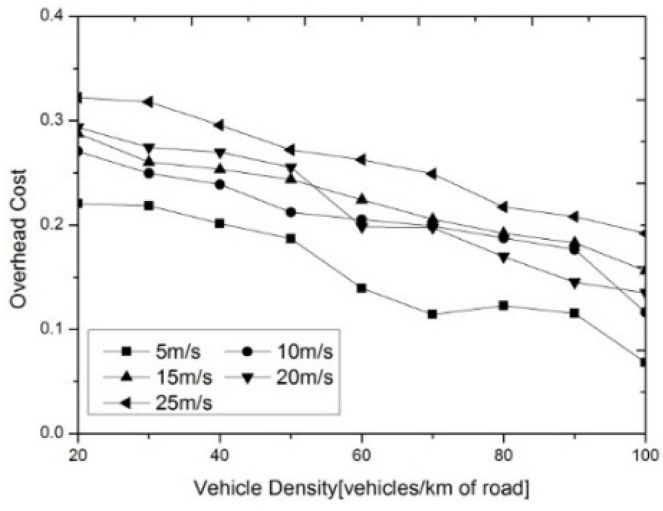
Overhead with different vehicle densities.

Figure 5 shows the overhead of MGADA with different vehicle densities and velocities. The overhead of MGADA declines with an increment in vehicle density when the maximum vehicle velocity is fixed. By contrast, when the vehicle density is fixed, the overhead rises with an increment in vehicle velocity, because the clusters in a VANET become more stable as the vehicle velocity decreases. In a denser and more stable network, the number of cluster reconstruction and message retransmission can be reduced, which in turn can also reduce the overhead of MGADA.

**Figure 6:**
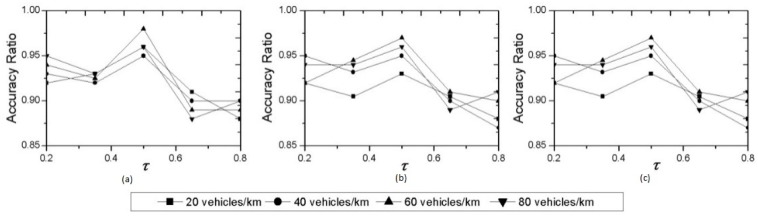
Accuracy ratio under different utility factors, vehicle densities and maximum vehicle velocities:(**a**) 5 m/s; (**b**) 15m/s; and (**c**) 25m/s.

A group of experiments are also conducted to observe the effect of utility factor on MGADA, under different vehicle velocities and densities. As shown in Figure 6, the accuracy ratio is distributed within [0.85, 1.00] when the utility factor varies from 0.2 to 0.8. The utility factor significantly influences aggregation performance. When the utility factor is too large, the weight of the network stability function is small, and it is difficult to maintain a relatively stable condition in a sub-cluster for a long period for the cluster head. When the utility factor is too small, the aggregation environment is sufficiently stable, and many packages are deleted in the aggregation process. Thus, aggregation performance is also insufficient. We also find that MGADA achieves the best performance in aggregation accuracy when the utility factor is 0.5, the maximum velocity is 5 m/s and vehicle density is 60 vehicles/km, as shown in Figure 6.

## 6. Conclusions

A cluster aggregation scheme with improved stability is proposed in this study. First, node sample qualities are presented to distinguish nodes from one another. The link divergence and intensity variation of the cluster structure are evaluated in detail. Moreover, the collaborative relations among the nodes are mapped for multi-players who compete against and cooperate with one another in the game. Finally, Nash equilibrium and the interruption process are utilized to achieve an optimal transmission strategy. MGADA works well in the simulation experiments. In the future, an efficient clustering algorithm will be explored and applied to our work to achieve data aggregation in large-scale VANETs.

## Figures and Tables

**Table 1:** Parameters settings used in the simulation.

Parameter	Remark, Default Value
Simulation time	1800 s
Area range	10,000 m × 10,000 m
Maximum speed	5, 10, 15, 20, 25 (m/s)
Vehicle Density	20, 40, 60, 80, 100 (vehicles/km)
Sampling period	30 s
*w*	Sliding window size, 10
*α*	Adjustment factor in Equation (2), 0.5
τ	Adjustment factor in Equation (14), 0.5
*R_trans_*	Maximum transmission range, 300 m
*R_nei_*	Neighborhood radius of cluster members, 100 m
Radio Propagation Model	Tow-ray ground

## Appendix A. Proof of the Existence of a Unique Nash equilibriumin MGADA

**Proof of Theorem 1.** The theorem that identifies the existence of Nash equilibrium is obtained from [32]. The first condition is satisfied because strategy space *p_i_* is a set composed of 0 and 1, that is, *p_i_* is a non-empty, convex, and compact subset of the same Euclidean space *R^n^*. We suppose that *Y_kx_* and *Z_x_*(*k*) represent the values of the separation vector gain from node x to in the *k*-th round and neighborhood retroaction quality of node *x* in the *k*-th clock period, respectively. These values can be expressed as follows:

(A1) $$Y_{kx}=Max\left(10\mathrm{lg}\frac{D^{\left(k\right)}\left(x\to H\right)}{D^{\left(k-1\right)}\left(x\to H\right)},0\right)$$

(A2) $$Z_{kx}=\sum_{y\in N_{x}^{\left(k\right)}}Max\left(\mathrm{lg}\frac{SQ_{x}^{\left(k\right)}}{SQ_{y}^{\left(k\right)}},0\right)$$

Suppose that *n* nodes are distributed in a certain area and make a sample for *T* clock periods, then:

(A3) $$s^{2}=\frac{x_{1}^{2}+x_{2}^{2}+...+x_{n}^{2}}{n}-\frac{{\left(x_{1}+x_{2}+...+x_{n}\right)}^{2}}{n^{2}}$$

Given that *Y* and *Z* are determined by the sample data, no correlation exists between variables *Y* and *Z*. By substituting Equations (A1)–(A3) into Equation (14), the following expression can be derived:

(A4) $$\begin{array}{ll} f\left(p_{1},p_{2},...,p_{n}\right) & =-\tau \times CRD_{P}^{\left(T_{2}\right)}+\left(\tau -1\right)\times CVD_{H}^{\left(T_{2}\right)}\\ & =-\frac{\tau }{n}\times \left(n-p_{1}Z_{T_{2}1}-p_{2}Z_{T_{2}2}-...-p_{n}Z_{T_{2}n}\right)\\ & +\frac{\left(\tau -1\right)}{T_{2}-q+1}\times \left(\begin{array}{l} \frac{1}{n}\left({\left(p_{1}Y_{q1}\right)}^{2}+{\left(p_{2}Y_{q2}\right)}^{2}+...+{\left(p_{n}Y_{qn}\right)}^{2}\right)\\ -\frac{1}{n^{2}}{\left(p_{1}Y_{q1}+p_{2}Y_{q2}+...+p_{n}Y_{qn}\right)}^{2} \end{array}\right)\\ & +\frac{\left(\tau -1\right)}{T_{2}-q+1}\times \left(\begin{array}{l} \frac{1}{n}\left({\left(p_{1}Y_{(q+1)1}\right)}^{2}+{\left(p_{2}Y_{(q+1)2}\right)}^{2}+...+{\left(p_{n}Y_{(q+1)n}\right)}^{2}\right)\\ -\frac{1}{n^{2}}{\left(p_{1}Y_{(q+1)1}+p_{2}Y_{(q+1)2}+...+p_{n}Y_{(q+1)n}\right)}^{2} \end{array}\right)\\ & +...\\ & +\frac{\left(\tau -1\right)}{T_{2}-q+1}\times \left(\begin{array}{l} \frac{1}{n}\left({\left(p_{1}Y_{T_{2}1}\right)}^{2}+{\left(p_{2}Y_{T_{2}2}\right)}^{2}+...+{\left(p_{n}Y_{T_{2}n}\right)}^{2}\right)\\ -\frac{1}{n^{2}}{\left(p_{1}Y_{T_{2}1}+p_{2}Y_{T_{2}2}+...+p_{n}Y_{T_{2}n}\right)}^{2} \end{array}\right) \end{array}$$

Suppose that *i* is not equal to *j*, that is, *i* ≠ *j*, then:

(A5) $$\frac{\partial f\left(p_{1},p_{2},...,p_{n}\right)}{\partial p_{u}\partial p_{v}}=\frac{2\left(1-\tau \right)}{n^{2}\left(T_{2}-q+1\right)}\sum_{i=q}^{T_{2}}Y_{iu}Y_{iv}\geq 0$$

Thus, $f\left(p_{1},p_{2},...,p_{n}\right)$ is a type of super modular function Thus, $f\left(p_{1},p_{2},...,p_{n}\right)$ has a unique Nash equilibrium solution. Given the reconstruction criterion presented in Subsection 4.5, the range of *Y_kx_* is [0, 10·lg3], that is, $Y_{kx}\in \left[0,10\mathrm{lg}3\right]$. In Equation (A2), the value of *Z_kx_* is larger than 0, that is, $Z_{kx}\geq 0$. Under this condition, that is, $Z_{kx}\geq 0$ and $Y_{kx}\in \left[0,10\mathrm{lg}3\right]$, there is one and only one solution $\left(p_{1}^{*},p_{2}^{*},...,p_{n}^{*}\right)$ that satisfies the Nash equilibrium condition, that is:

(A6) $$f\left(p_{1}^{*},...,p_{i-1}^{*},p_{i}^{*},p_{i+1}^{*},...,p_{n}^{*}\right)\geq f\left(p_{1}^{*},...,p_{i-1}^{*},p_{i},p_{i+1}^{*},...,p_{n}^{*}\right),p_{i}\in P$$

## Appendix B. Solution to Nash equilibrium

According to [35], we can obtain the Nash equilibrium solution using equations $\frac{\partial f\left(p_{1},p_{2},...,p_{n}\right)}{\partial p_{1}}=0$,…, $\frac{\partial f\left(p_{1},p_{2},...,p_{n}\right)}{\partial p_{n}}=0$, that is:

(B1) $$\left\{ \begin{array}{l} \frac{\tau }{n}Z_{T_{2}1}+\frac{2\left(\tau -1\right)p_{1}}{n\left(T_{2}-q+1\right)}\sum_{i=q}^{T_{2}}Y_{i1}^{2}+\frac{2\left(1-\tau \right)}{n^{2}\left(T_{2}-q+1\right)}\sum_{i=q}^{T_{2}}\sum_{j=1}^{n}p_{j}Y_{i1}Y_{ij}=0\\ \frac{\tau }{n}Z_{T_{2}2}+\frac{2\left(\tau -1\right)p_{2}}{n\left(T_{2}-q+1\right)}\sum_{i=q}^{T_{2}}Y_{i2}^{2}+\frac{2\left(1-\tau \right)}{n^{2}\left(T_{2}-q+1\right)}\sum_{i=q}^{T_{2}}\sum_{j=1}^{n}p_{j}Y_{i2}Y_{ij}=0\\ ...\\ \frac{\tau }{n}Z_{T_{2}n}+\frac{2\left(\tau -1\right)p_{n}}{n\left(T_{2}-q+1\right)}\sum_{i=q}^{T_{2}}Y_{in}^{2}+\frac{2\left(1-\tau \right)}{n^{2}\left(T_{2}-q+1\right)}\sum_{i=q}^{T_{2}}\sum_{j=1}^{n}p_{j}Y_{in}Y_{ij}=0 \end{array}\right.$$

In a certain sample instance, the values of *Y* and *Z* are known. Thus, the Nash equilibrium solution $\left(p_{1}^{*},p_{2}^{*},...,p_{n}^{*}\right)$ is available.
